# Optimization of insulin-like growth factor-1 supplementation enhances oocyte quality by modulating oxidative stress and apoptotic pathways during *in vitro* maturation of Kacang goat oocytes

**DOI:** 10.14202/vetworld.2025.3545-3560

**Published:** 2025-11-27

**Authors:** Widjiati Widjiati, Epy Muhammad Luqman, Ninik Darsini, Aulanni’am Aulanni’am, Wan Nor Fitri Bin Wan Jaafar, Suzanita Utama, Devia Yoanita Kurniawati, Zahra Shabira, Viski Fitri Hendrawan

**Affiliations:** 1 Department of Veterinary Anatomy, Faculty of Veterinary Medicine, Universitas Airlangga, Surabaya, Indonesia; 2 Department of Medical Biology, Faculty of Medicine, Universitas Airlangga, Indonesia; 3 Department of Biochemistry, Faculty of Veterinary Medicine, Universitas Brawijaya, Malang, Indonesia; 4 Department of Farm and Exotic Animal Medicine and Surgery, Faculty of Veterinary Medicine, Universiti Putra Malaysia, Serdang, Selangor, Malaysia; 5 Department of Veterinary Reproduction, Faculty of Veterinary Medicine, Universitas Airlangga, Surabaya, Indonesia; 6 Department of Veterinary Reproduction, Faculty of Veterinary Medicine, Universitas Brawijaya, Malang, Indonesia

**Keywords:** apoptosis, *in vitro* oocyte maturation, Insulin-like growth factor-1, Kacang goat, oocyte quality, oxidative stress, phosphatidylinositol 3-kinase/protein kinase B

## Abstract

**Background and Aim:**

Insulin-like growth factor-1 (IGF-1) plays a crucial role in folliculogenesis and oocyte maturation by regulating oxidative stress and apoptotic pathways. However, the optimal IGF-1 concentration for small ruminant oocytes, particularly the native Kacang goat, remains undefined. This study aimed to determine the optimal IGF-1 dose for improving oocyte quality during *in vitro* oocyte maturation (IVM) by evaluating oxidative stress and apoptosis markers.

**Materials and Methods:**

Ovaries (n = 120) were collected from local slaughterhouses, and cumulus–oocyte complexes were aspirated, selected, and randomly divided into four groups: Control (0 ng/mL IGF-1) and treatments with 50, 100, and 150 ng/mL IGF-1 supplementation. Mature oocytes were analyzed for oxidative stress biomarkers, including superoxide dismutase (SOD-1), glutathione (GSH), and malondialdehyde (MDA), using an enzyme-linked immunosorbent assay, and for apoptosis regulators, B-cell lymphoma 2-associated X protein (BAX), B-cell lymphoma-2 (BCL-2), and cytochrome c, using immunocytochemistry. Data were analyzed using one-way analysis of variance and Duncan’s *post hoc* test (p < 0.05).

**Results:**

IGF-1 supplementation produced concentration-dependent effects. The 100 ng/mL group (T2) exhibited the highest SOD-1 (2.07 ± 0.60) and GSH (8.07 ± 1.79) levels (p < 0.05), while MDA increased with higher IGF-1 doses, indicating a threshold beyond which oxidative stress is induced. Anti-apoptotic BCL-2 and cytochrome c expressions peaked at 50 ng/mL IGF-1 (10.73 ± 1.56 and 11.73 ± 0.99, respectively), whereas the pro-apoptotic marker BAX was lowest in the same group and increased at higher doses. The findings suggest that 50 ng/mL IGF-1 effectively maintains redox balance and mitochondrial stability through phosphatidylinositol 3-kinase/protein kinase B-mediated regulation.

**Conclusion:**

This study demonstrates, for the 1^st^ time, a dose-dependent, biphasic effect of IGF-1 on oxidative and apoptotic pathways in Kacang goat oocytes. An optimal concentration of 50 ng/mL IGF-1 enhances oocyte maturation by balancing antioxidant defense and anti-apoptotic mechanisms, whereas higher concentrations induce oxidative stress. These findings establish a breed-specific IVM optimization protocol that supports genetic preservation and sustainable reproductive biotechnology for indigenous goats.

## INTRODUCTION

Insulin-like growth factor 1 (IGF-1) is a conserved peptide hormone found in all species, including domestic and wild animals, as well as humans. The IGF-1 family plays vital roles in cellular growth, proliferation, and differentiation, with each member binding to specific receptors to initiate distinct intracellular metabolic pathways [1]. In ovarian folliculogenesis, IGF-1 is involved in multiple physiological processes, such as oogenesis, oocyte maturation, ovulation, luteal function, and follicular atresia. It modulates the action of follicle-stimulating hormone (FSH) on granulosa cells of antral follicles and acts synergistically with luteinizing hormone (LH) to enhance androgen biosynthesis in theca cells. Furthermore, oocytes themselves express IGF-1 messenger ribonucleic acid, which functions in both autocrine and paracrine modes to regulate the proliferation, differentiation, and steroidogenesis of granulosa and cumulus cells [2].

IGF-1 influences oocyte quality primarily through its regulation of apoptotic pathways, oxidative stress responses, and inflammatory mechanisms [3]. Supplementation of IGF-1 during *in vitro* oocyte maturation (IVM) has been shown to enhance oocyte competence and improve the success of oocyte banking programs [4]. Physiologically, IGF-1 is synthesized through two main mechanisms: (a) Hepatic production stimulated by pituitary-derived growth hormone (GH) through GH receptor activation, leading to increased IGF transcription and (b) local synthesis in peripheral reproductive tissues such as the oviduct and endometrium under the influence of GH and sex steroids to support follicular and oocyte development [5].

*In vitro* studies demonstrate that IGF-1 can inhibit granulosa cell (GC) apoptosis and stimulate proliferation, confirming its regulatory influence on GC function. However, excessive IGF-1 concentrations may upregulate apoptosis-related genes, thereby increasing the susceptibility of GC to cell death. For instance, IGF-1 at 10 ng/mL inhibits apoptosis in cultured bovine GCs, whereas higher concentrations (100 ng/mL) promote apoptosis, indicating a dose-dependent dual effect [6]. Apoptosis often follows oxidative stress arising from the accumulation of reactive oxygen species (ROS) within mitochondria, which activate apoptotic regulators, such as B-cell lymphoma 2 (BCL-2) and BCL-2-associated X protein (BAX), as well as heat shock proteins, along with pro-apoptotic genes such as caspase 3 (*CASP*-3) and caspase 6 (*CASP*-6). These changes disrupt the mitochondrial membrane potential, leading to the release of cytochrome c and the activation of downstream apoptotic cascades.

In response, oocytes activate transcription factors such as forkhead box O3 and Kelch-like erythroid cell-derived protein with CNC homology (ECH)-associated protein 1, which upregulate antioxidant genes including superoxide dismutase (SOD-1 1, SOD-1 2), glutathione (GSH) disulfide reductase, GSH S-transferase alpha 3, and catalase to counter oxidative stress [7, 8]. Heat shock proteins further function as molecular chaperones, managing stress-denatured proteins by activating the mitogen-activated protein kinase (MAPK) pathway and BAX, while inhibiting CASP-3. IGF-1 primarily exerts its anti-apoptotic effects by activating the phosphatidylinositol 3-kinase/protein kinase B (PI3K/AKT) signaling pathway, which upregulates the anti-apoptotic BCL-2 protein family. This signaling cascade stabilizes mitochondrial membrane integrity in oocytes and regulates the phosphorylation and inactivation of pro-apoptotic molecules such as BAX and BAK, preventing cytochrome c release and promoting oocyte survival [9].

Although the role of IGF-1 in mammalian folliculogenesis and oocyte maturation has been widely explored in cattle, pigs, and humans, its precise biochemical and molecular effects during IVM in goats remain poorly defined. Previous research has focused primarily on morphological maturation rates or cumulus expansion, often neglecting the molecular determinants of oocyte quality such as oxidative stress regulation and apoptotic signaling. Moreover, most reports have used bovine or porcine models to infer IGF-1-mediated cytoprotective mechanisms, assuming cross-species equivalence, despite clear differences in follicular microenvironments, receptor distribution, and redox metabolism between small ruminants and large livestock species.

Existing studies indicate that IGF-1 supplementation can enhance cytoplasmic maturation, mitochondrial function, and cumulus cell proliferation; however, contradictory results have been observed at varying concentrations. While low-to-moderate IGF-1 doses appear to promote granulosa-cell survival and antioxidant activity, supraphysiological levels have been linked to excessive ROS generation and pro-apoptotic activation. However, no comprehensive study has yet delineated the dose-dependent duality of IGF-1 on both oxidative balance and apoptosis regulation in caprine oocytes. In particular, the relationship between IGF-1-driven activation of the PI3K/AKT signaling cascade and downstream molecular indicators, SOD-1, GSH, malondialdehyde (MDA), BAX, BCL-2, and cytochrome c, remains unexplored in goat oocytes.

Furthermore, breed-specific investigations, especially in indigenous small-ruminant species such as the Kacang goat, are extremely limited. Given the breed’s high adaptation value and genetic significance for tropical livestock systems, understanding the biochemical mechanisms underpinning its oocyte competence is critical for developing optimized, cost-effective IVM protocols. Therefore, a clear knowledge gap exists regarding how graded IGF-1 concentrations modulate oxidative stress, mitochondrial stability, and apoptotic pathways in goat oocytes, as well as how these molecular responses translate into enhanced oocyte viability and developmental potential.

This study aimed to evaluate the dose-dependent effects of IGF-1 supplementation on oxidative stress and apoptotic regulation during *IVM* of caprine oocytes, integrating biochemical and immunocytochemical analyses to reveal mechanistic insights. Specifically, the objectives were:

1. To quantify oxidative stress biomarkers, SOD-1, GSH, and MDA, in oocytes mature under different IGF-1 concentrations (0, 50, 100, 150 ng/mL) to determine the optimal dose that enhances antioxidant defense while minimizing lipid peroxidation.
2. To assess the expression of apoptotic and anti-apoptotic markers (BAX, BCL-2, cytochrome c) through immunocytochemistry, elucidating how IGF-1 modulates mitochondrial-dependent apoptotic signaling.
3. To interpret the interrelationship between oxidative and apoptotic responses within the IGF-1/PI3K/AKT signaling framework and identify the concentration range that sustains redox homeostasis and oocyte survival.

By combining oxidative and apoptotic profiling, this research provides an integrated mechanistic model explaining how IGF-1 orchestrates cellular resilience during IVM. The findings are expected to define an optimal IGF-1 supplementation threshold for goat oocytes, improve the molecular quality of *in vitro*-mature gametes, and contribute to the refinement of assisted reproductive technologies (ARTs) for genetic conservation and productivity enhancement in small-ruminant species.

## MATERIALS AND METHODS

### Ethical approval

The experimental procedures involving animal biological materials were reviewed and approved by the Animal Care and Use Committee (ACUC), Faculty of Veterinary Medicine, Universitas Airlangga, Surabaya, Indonesia (Approval No. 1.KEH.025.02.2024; approved on February 25, 2024). All procedures strictly adhered to the institutional guidelines for the ethical use of animals in research and conformed to the principles outlined in the Animal Research: Reporting of *In Vivo* Experiments (ARRIVE 2.0) guidelines.

Since the study utilized abattoir-derived ovaries from animals slaughtered for commercial purposes, no live animals were sacrificed exclusively for research, and no additional ethical or welfare concerns were introduced beyond standard slaughterhouse practices.

The research team ensured minimal environmental impact, proper biosafety handling, and compliance with Indonesian animal welfare regulations, as outlined in Law No. 18/2009 and Government Regulation No. 95/2012, regarding livestock and animal health.

### Study period and location

This study was conducted over an 8-month period, from January to August 2024. The experimental workflow included three key stages: (1) Oocyte retrieval from slaughterhouse samples, (2) IVM of oocytes at the *in vitro* laboratory, Faculty of Veterinary Medicine, Universitas Airlangga, and (3) biochemical and immunochemical analyses. The oxidative stress markers were detected using enzyme-linked immunosorbent assay (ELISA) (Shanghai Korain Biotech Co., Ltd. Bioassay Technology Laboratory [BT LAB], Shanghai, China) kits at the Faculty of Science, Universitas Airlangga, whereas the immunocytochemistry analysis was performed at the Pathology Laboratory, Faculty of Veterinary Medicine, Universitas Airlangga, Surabaya.

### Collection and transportation of ovaries

A total of 120 ovaries were collected from healthy adult female Kacang goats (aged 6–12 months) at a local slaughterhouse. Only ovaries from visibly non-pregnant animals were used, and estrous cycle stages were not determined. Ovaries were collected between 05:00 and 06:00 AM and transported to the *in vitro* laboratory within 2 h in an insulated thermal flask (Akebonno, Jakarta, Indonesia) containing warm (37°C–38°C) 0.9% saline (NaCl) supplemented with streptomycin. Upon arrival, the ovaries were rinsed 3 times with sterile, pre-warmed phosphate-buffered saline (PBS) to remove blood and debris [10].

### Oocyte retrieval and selection

Follicles were aspirated using a 10 mL syringe fitted with an 18G needle (Onemed, PT Jayamas Medical Industry Tbk, Sidoarjo, Indonesia) containing 1 mL sterile PBS. Typically, aspiration of 10–20 ovaries yielded approximately 80–100 cumulus–oocyte complexes (COCs). The aspirated follicular fluid was examined under an Olympus X41 inverted microscope (Evident Scientific, Tokyo, Japan) and COCs were identified and graded based on morphological quality. Only oocytes with three or more layers of compact cumulus cells and a homogeneous, evenly granulated ooplasm were selected for use. Oocytes showing incomplete cumulus layers, denudation, or atretic signs (e.g., darkened or vacuolated cytoplasm) were excluded from the study. The experiments were conducted in three independent biological replicates, with each replicate performed on different days using newly collected ovaries to ensure biological variability [10, 11].

### IVM protocol

Selected COCs were washed and cultured in TCM-199 medium (Thermo Fisher Scientific, Waltham, MA, USA) supplemented with 10% fetal bovine serum (FBS) (Cat. No. 12103C; Sigma-Aldrich, St. Louis, MO, USA), 10 μg/mL FSH, 10 μg/mL LH, and 1% penicillin–streptomycin. The oocytes were randomly allocated into four experimental groups:

- Control: Base medium without IGF-1 supplementation
- T1: 50 ng/mL IGF-1
- T2: 100 ng/mL IGF-1
- T3: 150 ng/mL IGF-1

Each group contained 10–15 oocytes placed in 30 μL microdroplets of maturation medium under mineral oil and incubated for 22 h at 38.5°C, with 5% CO_2_ and >95% relative humidity. The selected IGF-1 concentrations were based on previous studies in domestic species showing biphasic, concentration-dependent effects of IGF-1 on follicular and oocyte physiology [8, 10, 12, 13].

Oocyte nuclear maturation was evaluated by the extrusion of the first polar body, indicating transition to the metaphase II stage, using an Olympus X41 inverted microscope.

### Assessment of oxidative stress markers

Following IVM, oocytes were collected for biochemical analysis. For each experimental condition, 10 replicates were prepared, with each replicate consisting of 10 pooled oocytes. The oocytes were lysed in cell lysis buffer, incubated for 30 min, vortexed for 15 min, and centrifuged at 489 × *g* for 10 min. The supernatant was used to determine the levels of SOD-1, GSH, and MDA using commercial ELISA kits (Shanghai Korain Biotech Co., Ltd. [BT LAB]).

- Cu-Zn SOD-1-1 ELISA Kit (Cat. No. E1444Ra)
- MDA ELISA Kit (Cat. No. E0198Bo)
- GSH ELISA Kit (Cat. No. EA0113Ra)

According to the manufacturer’s datasheets, these antibodies demonstrated cross-reactivity with caprine targets due to conserved protein homology. Each treatment replicate was considered a technical replicate, originating from the same oocyte pool. Concentration values were derived from standard curves and expressed per pooled sample rather than per individual oocyte [10].

### Apoptotic marker assessment (immunocytochemistry)

For immunocytochemical evaluation, five replicates were prepared for each experimental group. Each replicate consisted of 10 mature oocytes mounted on poly-L-lysine-coated slides and sealed beneath a petroleum jelly coverslip. Samples were fixed for 24 h, then treated sequentially with 3% hydrogen peroxide (H_2_O_2_), rinsed in PBS, and digested with 0.025% trypsin at 37°C. After washing, an Ultra-V block was applied to prevent non-specific binding. Slides were incubated for 60 min with the following primary antibodies (Santa Cruz Biotechnology, Dallas, TX, USA):

- Cytochrome c (A-8: sc-13156, 1:200)
- BAX (B-9: sc-7480, 1:200)
- BCL-2 (C-2: sc-7382, 1:200)

Negative controls (without primary antibody) were included in each run to confirm staining specificity. The following day, slides were washed and incubated with a biotinylated secondary antibody for 60 min, followed by sequential incubation with biotinylated links and streptavidin. The chromogenic reaction was developed using 3,3′-diaminobenzidine, and counterstaining was performed with methylene green (Thermo Fisher Scientific). Slides were examined under an Olympus CX-41 microscope (Evident Scientific) at 400× magnification, following the manufacturer’s protocol [14].

Protein expression for BAX, BCL-2, and cytochrome c was semi-quantitatively scored using the immunoreactive score (IRS), calculated by multiplying the percentage of positively stained cells by staining intensity. Each replicate was treated as a technical replicate for analytical consistency.

### Statistical analysis

All quantitative data were analyzed using the Statistical Package for the Social Sciences Software version 2023 (IBM Corp., NY, USA), with p < 0.05 considered statistically significant. The experimental unit for all analyses was the replicate (pooled oocyte sample). Data normality was verified using the Shapiro–Wilk test, and variance homogeneity was assessed using Levene’s test. Parametric data were analyzed using one-way analysis of variance followed by Duncan’s multiple range test for *post hoc* comparisons. Non-parametric data were evaluated using the Kruskal–Wallis test, followed by the Mann–Whitney U test for pairwise group comparisons.

## RESULTS

### Oxidative stress analysis (ELISA)

#### Experimental overview

**Figure 1:**
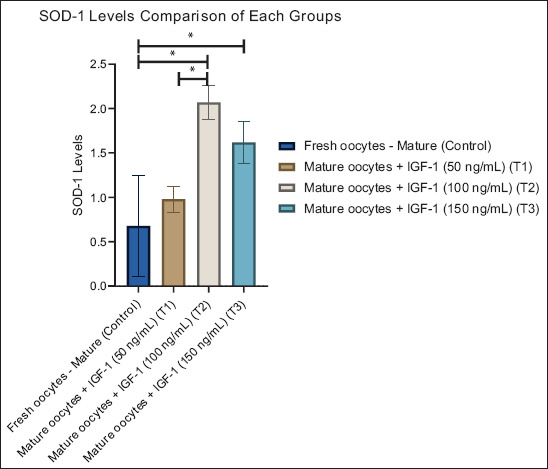
Graphic visualization of the superoxide dismutase-1-1 levels of each group. Data are presented as the mean ± standard error of the mean (n = 10 replicates/group). (*) = Represents statistically significant differences (p < 0.05) between the group bars.

**Figure 2:**
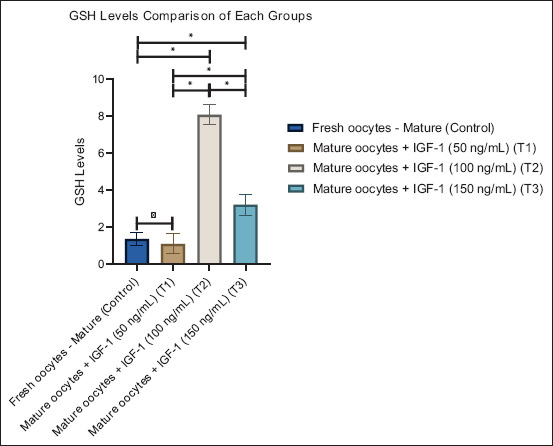
Graphic visualization of the glutathione levels of each group. Data are presented as the mean ± standard error of the mean (n = 10 replicates/group). (*) = Represents statistically significant differences (p < 0.05) between the group bars.

**Figure 3:**
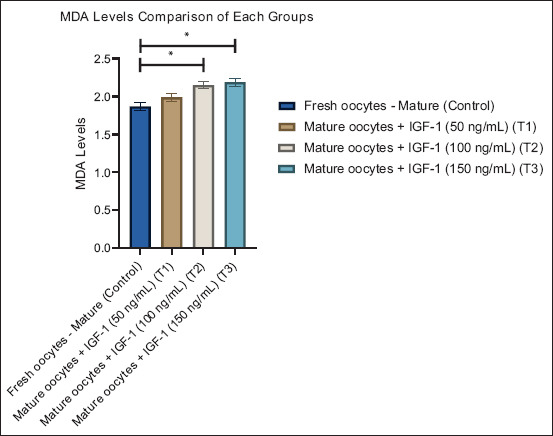
Graphic visualization of the malondialdehyde levels of each group. Data are presented as the mean ± standard error of the mean (n = 10 replicates/group). (*) = Represents statistically significant differences (p < 0.05) between the group bars.

The experimental design consisted of one control group (CG) (no IGF-1 supplementation) and three treatment groups supplemented with 50 ng/mL (T1), 100 ng/mL (T2), and 150 ng/mL (T3) IGF-1. This investigation aimed to determine how IGF-1 affects the oxidative balance in mature oocytes by evaluating three biomarkers of oxidative stress, SOD-1-1, GSH, and MDA. These parameters were analyzed to elucidate both the protective and potential pro-oxidant effects of IGF-1 supplementation. Tables 1 and 2 and Figures 1–3 summarize the obtained results.

**Table 1:** Comparison of oxidative stress markers (SOD-1, GSH, and MDA) in each experimental *in vitro* oocyte maturation group.

Groups	n	SOD-1-1 levels	GSH levels	MDA levels
Fresh oocytes: Mature (control)	10	0.68 ± 1.80^a^	1.36 ± 1.09^a^	1.87 ± 0.18^a^
Mature oocytes + IGF-1 (50 ng/mL) (T1)	10	0.98 ± 0.46^ab^	1.09 ± 1.71^b^	1.99 ± 0.16^ab^
Mature oocytes + IGF-1 (100 ng/mL) (T2)	10	2.07 ± 0.60^c^	8.07 ± 1.79^c^	2.15 ± 0.15^b^
Mature oocytes + IGF-1 (150 ng/mL) (T3)	10	1.62 ± 0.75^bc^	3.21 ± 1.80^d^	2.19 ± 0.16^b^

*Different superscripts in the same column indicate a significant difference (p < 0.05). SOD = Superoxide dismutase, MDA = Malondialdehyde, GSH = Glutathione, IGF-1 = Insulin-like growth factor-1.

**Table 2:** Comparison of apoptosis regulator (BAX, BCL-2, and cytochrome c) expression scores in each experimental *in vitro* oocyte maturation group.

Groups	n	BAX	BCL-2	Cytochrome c
Fresh oocytes: Mature (control)	5	1.87 ± 0.18^a^	4.00 ± 2.36^a^	4.33 ± 4.98^ab^
Mature oocytes + 50 ng/mL IGF-1 (T1)	5	1.99 ± 0.16^ab^	10.73 ± 1.56^b^	11.73 ± 0.99^b^
Mature oocytes + IGF-1 (100 ng/mL) (T2)	5	2.15 ± 0.15^b^	10.73 ± 1.56^b^	10.00 ± 3.34^a^
Mature oocytes + IGF-1 (150 ng/mL) (T3)	5	2.19 ± 0.16^b^	2.13 ± 1.70^a^	6.20 ± 0.95^b^

*Different superscripts in the same column indicate a significant difference (p < 0.05). BAX = B-cell lymphoma 2-associated X protein, BCL-2 = B-cell lymphoma-2, IGF-1 = Insulin-like growth factor-1.

#### SOD-1-1 activity

The SOD-1-1 levels showed a clear dose-dependent increase following IGF-1 supplementation (Table 1). The CG displayed the lowest SOD-1-1 activity (0.68 ± 1.80), representing basal antioxidant capacity in the absence of IGF-1. A moderate rise was observed at 50 ng/mL (T1: 0.98 ± 0.46), while a significant increase occurred at 100 ng/mL (T2: 2.07 ± 0.60; p < 0.05). The activity slightly decreased at 150 ng/mL (T3: 1.62 ± 0.75), suggesting a plateau or threshold beyond which additional IGF-1 does not enhance antioxidant capacity. These findings demonstrate that IGF-1 improves enzymatic antioxidant defense, with the optimal effect at 100 ng/mL.

Figure 1 visually confirms this pattern, showing a progressive increase in SOD-1-1 activity from the control to T2, followed by a decline at T3. The graph illustrates a dose-dependent relationship and highlights the importance of precise IGF-1 dosage in maintaining redox stability.

#### GSH levels

GSH, a major non-enzymatic antioxidant, exhibited a non-linear response to IGF-1 treatment (Table 1). Control oocytes showed low GSH concentrations (1.36 ± 1.09). A minor decrease occurred at 50 ng/mL (T1: 1.09 ± 1.71), followed by a significant rise at 100 ng/mL (T2: 8.07 ± 1.79; p < 0.05). At 150 ng/mL (T3), GSH levels declined (3.21 ± 1.80) but remained above the control value. The pronounced peak at 100 ng/mL suggests that moderate IGF-1 concentrations enhance GSH synthesis or recycling, thus optimizing the non-enzymatic antioxidant defense system.

Figure 2 further illustrates this pattern. The bar representing T2 shows the highest GSH value, confirming that 100 ng/mL IGF-1 yields the greatest antioxidant benefit. The decrease at 150 ng/mL suggests that higher IGF-1 doses may impair GSH metabolism or increase oxidative requirements, reinforcing the need for precise dosage.

#### MDA concentrations

MDA, an indicator of lipid peroxidation, increased slightly but significantly with IGF-1 supplementation (Table 1). The CG displayed the lowest MDA value (1.87 ± 0.18), indicating minimal lipid damage under basal conditions. T1 showed a mild increase (1.99 ± 0.16), while higher levels were recorded in T2 (2.15 ± 0.15) and T3 (2.19 ± 0.16). Statistical differences (p < 0.05) suggest that elevated IGF-1 doses may induce mild oxidative stress, likely reflecting increased cellular metabolic activity. These results indicate a delicate balance between antioxidant activation and oxidative burden.

Figure 3 corroborates the tabulated data, showing a gradual upward trend in MDA with increasing IGF-1 concentration. The marked rise in T2 and T3 indicates that excessive supplementation can lead to elevated lipid peroxidation, emphasizing the need to maintain IGF-1 within physiological limits to preserve oocyte quality.

### Analysis of apoptotic factors (immunocytochemistry)

#### Overview

**Figure 4:**
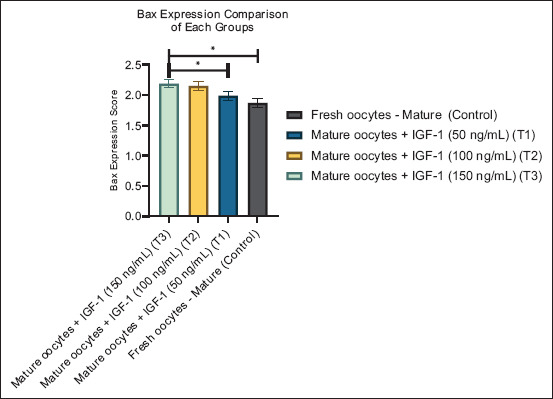
Visualization of the B-cell lymphoma 2-associated X protein expression score comparison of each group. Data are presented as the mean ± standard error of the mean (n = 5 replicates/group). (*) = Represents statistically significant differences (p < 0.05) between the group bars.

**Figure 5:**
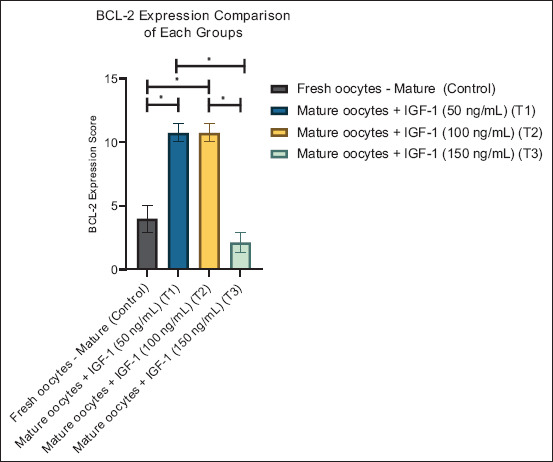
Graphic visualization of the B-cell lymphoma-2 expression score comparison of each group. Data are presented as the mean ± standard error of the mean (n = 5 replicates/group). (*) = Represents significant differences between the groups.

**Figure 6:**
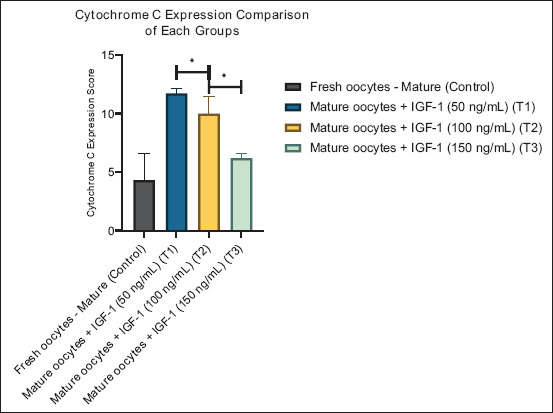
Graphic visualization of the cytochrome c expression score comparison of each group. Data are presented as the mean ± standard error of the mean (n = 5 replicates/group). (*) = Represents significant differences between the groups.

**Figure 7:**
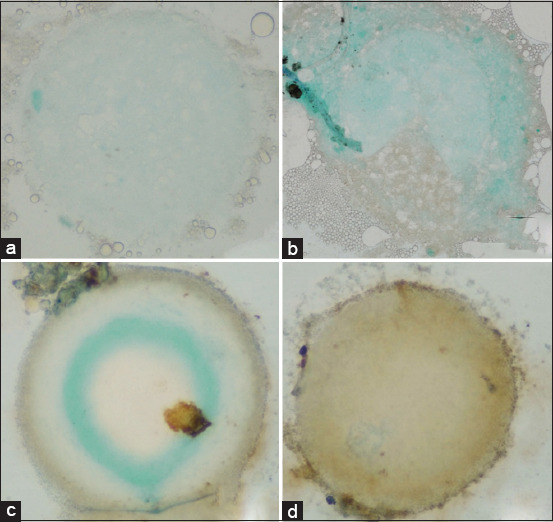
Representative immunohistochemical expression of Bax in Mature goat oocytes. Positive protein expression is visualized as a brown 3,3′-diaminobenzidine deposit, contrasted against the methylene green counterstain. (a) Control group, (b) T1 (50 ng/mL), (c) T2 (100 ng/mL), and (d) T3 (150 ng/mL). All images were captured at 400× magnification.

**Figure 8:**
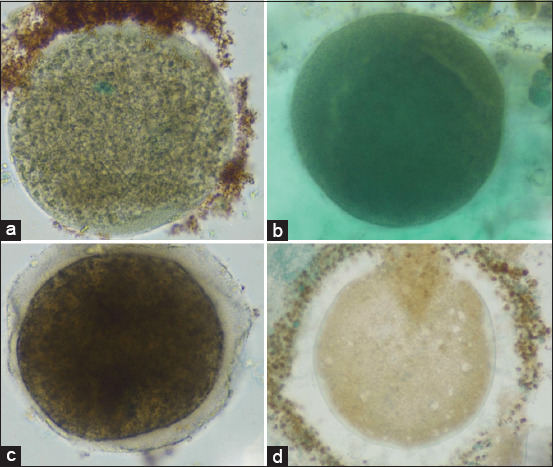
Representative immunohistochemical expression of B-cell lymphoma-2 in mature goat oocytes. The brown 3,3′-diaminobenzidine chromogen indicates a positive immunoreactive reaction for the protein, while non-reactive areas are counterstained with methylene green. (a) Control group, (b) T1 (50 ng/mL), (c) T2 (100 ng/mL), and (d) T3 (150 ng/mL). All images were captured at 400× magnification.

**Figure 9:**
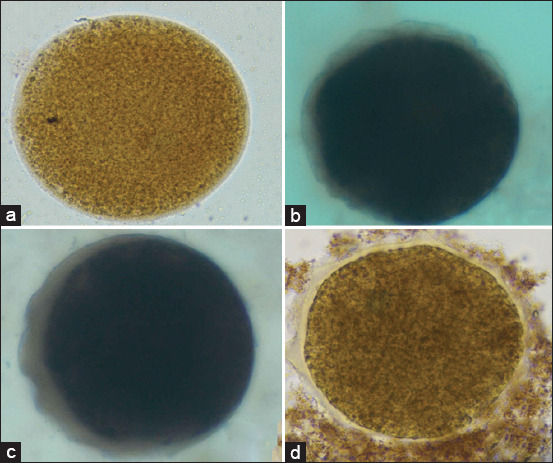
Representative immunohistochemical expression of cytochrome C in mature goat oocytes. A positive immunoreactive reaction is indicated by the brown chromogen 3,3′-diaminobenzidine deposit, while cellular structures are counterstained with methylene green. (a) Control group, (b) T1 (50 ng/mL), (c) T2 (100 ng/mL), and (d) T3 (150 ng/mL). All images were captured at 400× magnification.

The apoptotic response was assessed by measuring the expression of three key regulators: The pro-apoptotic protein BAX, the anti-apoptotic protein BCL-2, and cytochrome c, a mitochondrial marker of apoptotic activity. This analysis was designed to determine the IGF-1 concentration that best maintains mitochondrial integrity and minimizes apoptosis. The results are summarized in Table 2 and Figures 4–9.

#### BAX expression

BAX expression increased progressively with higher IGF-1 concentrations (Table 2). The CG exhibited the lowest level (1.87 ± 0.18), while a mild rise was observed in T1 (1.99 ± 0.16). The highest expressions were detected in T2 (2.15 ± 0.15) and T3 (2.19 ± 0.16), with statistically significant differences (p < 0.05). These data indicate that while IGF-1 supports maturation, excessive levels may activate pro-apoptotic pathways and compromise oocyte viability.

Figure 4 clearly depicts this trend, showing a steady increase from control to T3. The visualization highlights the importance of balanced IGF-1 supplementation to prevent apoptosis while promoting cell maturation.

#### BCL-2 expression

BCL-2, an anti-apoptotic marker, showed the opposite pattern (Table 2). Expression significantly increased in T1 and T2 (10.73 ± 1.56; p < 0.05), but markedly declined in T3 (2.13 ± 1.70), even below control levels (4.00 ± 2.36). This finding suggests that moderate IGF-1 concentrations promote survival signaling, whereas high concentrations compromise anti-apoptotic defense.

Figure 5 supports this observation, with the tallest bars at T1 and T2, followed by a sharp decline at T3. This inverse relationship highlights the importance of maintaining IGF-1 within an optimal range to sustain oocyte viability.

#### Cytochrome c expression

Cytochrome c expression, indicative of mitochondrial efficiency, was highest at moderate IGF-1 levels (T1: 11.73 ± 0.99; T2: 10.00 ± 3.34), both of which were significantly higher than the CG (4.33 ± 4.98). However, at 150 ng/mL (T3), cytochrome c levels decreased to 6.20 ± 0.95 (p < 0.05), reflecting reduced mitochondrial performance or enhanced oxidative stress at elevated IGF-1 concentrations.

Figure 6 shows a similar pattern, with a distinct peak at T1 and a subsequent decline at higher doses, which reinforces the notion that moderate IGF-1 supplementation enhances mitochondrial stability.

#### Immunoreactivity patterns

Immunocytochemical staining revealed characteristic brown coloration, indicating positive antibody–antigen reactions.

- Cytochrome c: Dark brown staining was evident in T1 and T2 groups, confirming higher mitochondrial activity after moderate IGF-1 supplementation.
- BAX: Gradual intensification of brown color from control to T3 indicated increasing BAX expression with rising IGF-1 levels, consistent with dose-dependent pro-apoptotic activation.
- BCL-2: More pronounced brown coloration was observed in the control and T1 groups than in T2 and T3, signifying stronger anti-apoptotic expression at lower IGF-1 doses.

These staining patterns (Figures 7–9) validate the quantitative findings, demonstrating that low-to-moderate IGF-1 concentrations enhance mitochondrial protection and anti-apoptotic signaling, whereas higher doses shift the balance toward oxidative and apoptotic stress.

## DISCUSSION

### Overview of experimental findings

Based on the experimental results, IGF-1 supplementation of caprine oocyte maturation media elicited diverse responses across treatment groups and measured parameters, potentially influencing oocyte developmental competence. The strength of this study lies in its integrated mechanistic framework, offering a deeper understanding of oocyte physiology beyond traditional morphological endpoints. Analyzing oxidative stress markers (SOD-1, GSH, and MDA) and apoptosis markers (BAX, BCL-2, and cytochrome c) allows us to connect the cellular antioxidant response to the regulation of programmed cell death pathways. This approach provides a clearer understanding of the effects of IGF-1 supplementation on oocyte quality and developmental competence, aiding in the optimization of IVM protocols.

### Evaluation of oxidative and apoptotic biomarkers

Several key biomarkers implicated in oxidative stress and apoptotic cascades, including SOD-1-1, MDA, GSH, BAX, BCL-2, and cytochrome c, were evaluated. These molecular indicators regulate cellular redox homeostasis and programmed cell death pathways, which significantly impact oocyte viability and developmental potential. The observed differential expression patterns of these proteins across the treatment groups suggest a complex interplay between IGF-1 concentration and cellular stress response mechanisms. Perturbations in the delicate balance between these molecular factors may induce oxidative stress, leading to the initiation of apoptotic signaling cascades [15]. Such intracellular milieu alterations can compromise oocyte quality, ultimately resulting in developmental arrest or cell death. This study underscores the nuanced effects of growth factor supplementation on oocyte physiology, highlighting the importance of the precise modulation of culture conditions to optimize IVM protocols for enhanced oocyte developmental competence [15, 16].

### Treatment-specific responses

Overall, treatment T1 appeared to be the most effective in upregulating BCL-2 and cytochrome c expression, whereas T3 exerted the strongest influence on BAX upregulation. These varied expression patterns suggest that each treatment possesses distinct mechanisms of action or induces differential levels of cellular stress, thereby modulating apoptotic pathways in unique ways. This observation underscores the complexity of cellular responses to various treatments and highlights the potential for targeted manipulation of the apoptotic process through specific interventions [8, 9].

### Mechanism of oxidative stress

Oxidative stress represents a state of physiological imbalance characterized by disequilibrium between pro-oxidant entities, primarily ROS, and cellular antioxidant defense mechanisms [16]. This condition occurs when ROS production exceeds the neutralizing capacity of endogenous antioxidant systems within a given cell, tissue, or biological fluid. A delicate homeostasis exists between the oxidative and reductive processes under normal physiological conditions, allowing for the controlled occurrence of essential biological oxidation reactions. However, this equilibrium can be disrupted by excessive ROS generation or a compromised antioxidant defense system [17]. The resultant imbalance leads to the unregulated oxidation of cellular substrates, potentially inducing a cascade of pathological alterations at the molecular and cellular levels. ROS overproduction can stem from various endogenous and exogenous factors, including mitochondrial dysfunction, environmental stressors, and metabolic perturbations. Conversely, the antioxidant defense system, comprising both enzymatic and non-enzymatic components, may be overwhelmed or function inadequately, further intensifying the oxidative burden. This redox imbalance can precipitate oxidative damage to crucial biomolecules, such as lipids, proteins, and nucleic acids, potentially compromising cellular function and integrity. Oxidative stress has been implicated in the pathogenesis of numerous diseases and is recognized as a significant contributor to cellular senescence and aging [17, 18].

### Mechanism of apoptosis

Apoptosis, colloquially referred to as “programmed cell death,” is a highly regulated physiological process observed in multicellular organisms. Various intrinsic and extrinsic factors trigger this sophisticated cellular mechanism that facilitates the systematic elimination of compromised or superfluous cells. The apoptotic cascade is characterized by a series of distinct morphological and biochemical alterations, including cellular volume reduction, nuclear DNA fragmentation, chromatin condensation, dynamic plasma membrane blebbing, and extracellular matrix detachment. This meticulously orchestrated cellular demise is a crucial homeostatic mechanism that controls the removal of damaged, infected, and potentially harmful cells, thereby maintaining tissue integrity and organismal health [17, 19]. This process is fundamentally distinct from necrosis because it does not typically elicit an inflammatory response. Instead, phagocytes efficiently engulf apoptotic cells, preventing the release of cellular contents into the surrounding tissue microenvironment. The intricate molecular machinery governing apoptosis involves a complex interplay of pro- and anti-apoptotic factors that are carefully balanced to ensure appropriate cellular responses to various stimuli. This tightly regulated process plays a pivotal role in embryonic development, tissue homeostasis, and immune system function, underscoring its significance in maintaining organismal health and preventing dysregulated cell death-associated pathological conditions [20, 21].

### Mitochondrial involvement in oxidative–apoptotic interactions

The intricate relationship between oxidative stress and apoptotic pathways in oocytes was elucidated by the pivotal role of mitochondria in this interconnected cellular process. Mitochondria, which primarily serve as the cell’s energy powerhouse, are also a significant source of ROS due to electron leakage from the respiratory chain [18]. The proximity of ROS to mitochondrial DNA, which is predominantly associated with the inner mitochondrial membrane’s matrix-facing side, creates a conducive environment for ROS-mediated mitochondrial DNA-protein crosslinking [18, 21]. This phenomenon initiates a cascade of harmful events, including enhanced mitochondrial fission and mitochondrial DNA damage exacerbation. Consequently, these alterations lead to a decline in ATP production and disruption of meiotic spindle integrity. Oxidative stress-induced changes in the mitochondrial membrane potential facilitate the release of cytochrome c from the mitochondria, a critical event in the activation of the apoptotic cascade [21, 22].

### Cellular signaling in oxidative–apoptotic regulation

The cellular response to this oxidative insult involves the modulation of various signaling pathways. Specifically, activation of the PI3K/AKT/mammalian target of rapamycin pathway or inhibition of nuclear factor kappa-light-chain-enhancer of activated B cells signaling can influence the levels of oxidative products, such as MDA, H_2_O_2_, and ROS. The released cytochrome c interacts with apoptotic protease-activating factor 1, triggering the activation of caspase 9 (CASP-9) and CASP-3, which are the key executioners of the apoptotic program [21, 23]. Complementary research has demonstrated that this process is accompanied by upregulation of cleaved caspase-3 and an increase in the BAX/BCL-2 ratio, both indicators of enhanced apoptotic activity. Concurrently, antioxidant enzymes, including SOD-1-2 and GSH peroxidase (GSH-Px), are downregulated, further compromising cells’ ability to counteract oxidative stress [23, 24]. This complex interplay among mitochondrial dysfunction, oxidative stress, and apoptotic signaling underscores the oocyte’s vulnerability to oxidative damage and underscores the critical importance of maintaining redox homeostasis to preserve oocyte quality and developmental potential [24].

### Functional role of IGF-1 in cellular regulation

IGF-1 orchestrates myriad cellular responses, including cell proliferation, survival, and metabolism. The PI3K/AKT pathway has been implicated in the upregulation of antioxidant defense mechanisms and the promotion of cell survival under oxidative stress [9]. The MAPK pathway contributes to gene expression regulation and cellular adaptation to environmental stress. The elucidation of these molecular mechanisms provides valuable insights into the potential therapeutic applications of IGF-1 in mitigating oxidative damage and promoting cellular resilience in various physiological and pathological contexts [25].

### Effects of IGF-1 concentrations on oxidative markers

In our investigation of the effects of IGF-1 supplementation at varying concentrations, we observed a dose-dependent elevation in MDA levels. The MDA concentration increased in the T1 and T3 groups but not in the T2 group. Although IGF-1 possesses antioxidant properties, our findings suggest that supraphysiological doses may paradoxically induce oxidative stress. This phenomenon can be attributed to the saturation of endogenous antioxidant defense mechanisms, leading to increased lipid peroxidation [26]. An alternative hypothesis posits that elevated IGF-1 concentrations may stimulate cellular metabolic activity, leading to ROS production that exceeds the antioxidant system’s neutralizing capacity [27].

### Regulation of SOD-1 activity

SOD-1, a critical antioxidant metalloenzyme, plays a pivotal role in cellular defense against oxidative stress by catalyzing the dismutation of superoxide anion radicals into molecular oxygen and (H_2_O_2_) [25]. Our experimental findings demonstrated a non-linear dose–response relationship between IGF-1 supplementation and SOD-1 activity. The highest SOD-1 levels were observed in the T2 group. This peak in SOD-1 activity at an intermediate IGF-1 dose suggests the existence of an optimal concentration for enhancing cellular antioxidant capacity. The observed bell-shaped response curve suggested a complex interplay between IGF-1 signaling and the regulation of antioxidant enzyme expression or activity. This phenomenon may be attributed to the activation of specific intracellular signaling cascades, such as the PI3K/AKT pathway, which has been implicated in the modulation of antioxidant gene expression [9, 23, 24]. The decline in SOD-1 activity at higher IGF-1 concentrations could be due to negative feedback mechanisms or the induction of cellular stress responses that counteract the initial antioxidant effects [28].

### GSH as a determinant of oocyte quality

GSH, a ubiquitous tripeptide thiol, plays a crucial role in determining the quality of *in vitro*-mature oocytes and their subsequent developmental potential. The intracellular GSH concentration is a critical indicator of oocyte cytoplasmic maturation, reflecting the capacity of the cell to maintain redox homeostasis and mitigate oxidative stress [21, 22]. As a potent low-molecular-weight antioxidant, GSH functions as a key regulator of intracellular redox equilibrium, providing the first line of defense against ROS and other oxidative stressors. The cytoprotective properties of GSH are attributed to its ability to scavenge free radicals, reduce peroxides, and participate in the regeneration of other antioxidants, thereby preserving cellular integrity and function [23]. Our analysis revealed a notable peak in GSH concentrations in the T2 group, which coincided with the optimal GSH concentration and the maximal SOD-1 activity observed at the same IGF-1 dose, suggesting a synergistic enhancement of the cellular antioxidant defense system. The concomitant elevation of both GSH and SOD-1 levels at this specific IGF-1 concentration indicates a potentially optimal redox environment for maintaining oocyte quality and viability [12]. As elucidated by Morohaku *et al*. [29], increased GSH levels play a critical role in ROS detoxification and in the preservation of oocyte developmental competence. The observed peak in GSH concentration in the T2 group suggests that this dose may provide an ideal balance between growth factor stimulation and antioxidant protection. Interestingly, we also observed a substantial decline in GSH levels (321.62 ± 180.22 arbitrary units) at the highest IGF-1 concentration (150 ng/mL, T3). This phenomenon can be attributed to two potential mechanisms. Supraphysiological IGF-1 levels may induce oxidative stress, leading to the depletion of intracellular GSH reserves. Elevated IGF-1 concentrations can stimulate cellular metabolic activity, leading to an increased oxidative burden that overwhelms the GSH-dependent antioxidant system [22].

### IGF-1 regulation of antioxidants and apoptotic proteins

IGF-1 upregulates antioxidants, such as GSH and GSH-Px [30]. GSH production is also influenced by IGF-1 through the PI3K/AKT pathway. IGF-1 signaling increases the expression of gamma-glutamylcysteine synthetase (γ-GCS), the rate-limiting enzyme in GSH synthesis, thereby maintaining cellular redox balance and protecting oocytes from oxidative damage during maturation [20]. However, our results indicate that high doses of IGF-1 may overstimulate cellular metabolism and mitochondrial activity, leading to increased ROS production, lipid peroxidation, and higher MDA levels [21]. IGF-1 plays a crucial role in regulating the expression of the proapoptotic protein BAX. IGF-1 can downregulate BAX expression, thereby inhibiting apoptotic cascades. In addition, IGF-1 upregulates BCL-2 expression, an anti-apoptotic protein that helps maintain mitochondrial membrane integrity and mitigate the efflux of pro-apoptotic factors from mitochondria to the cytosol. Thus, IGF-1 inhibits apoptosis by downregulating BAX and potentiating BCL-2 [31].

### Observed expression patterns of BAX, BCL-2, and cytochrome c

Our results showed that the highest BAX levels were found in the T3, T2, T1, and CGs; in contrast, the highest BCL-2 and cytochrome c levels were found in the T1 group. Interaction with the tyrosine kinase IGF-1 receptor (IGF-1R) initiates the cellular effects of IGF-1. IGF-1 binding induces IGF-1R autophosphorylation at tyrosine residues, facilitating the recruitment and activation of adaptor proteins, notably insulin receptor substrates. IRS activation propagates intracellular signaling cascades, including the PI3K/AKT and Ras/MAPK pathways [32]. The PI3K/AKT pathway may be the primary mediator of IGF-1 signaling. Activated PI3K catalyzes the conversion of phosphatidylinositol 4,5-bisphosphate to phosphatidylinositol 3,4,5-trisphosphate, which subsequently recruits and activates AKT. Phosphorylated AKT directly suppresses BAX expression through the phosphorylation and inactivation of BAX-inducing transcription factors [9]. Furthermore, AKT reduces BAX protein stability and impedes BAX oligomerization in the mitochondrial membrane. AKT also enhances BCL-2 expression and stability by increasing its transcriptional activity [21]. BCL-2 sequesters BAX and other pro-apoptotic proteins, such as Bak, thereby preventing the release of mitochondrial cytochrome c [33, 34].

### CASP regulation and anti-apoptotic mechanisms

The phosphorylated form of AKT plays a crucial role in inhibiting the activation of key apoptotic enzymes, particularly CASP-9 and CASP-3 [23]. Through phosphorylation, AKT modifies the pro-apoptotic protein - Bcl-2-associated death promoter (BAD), causing it to associate with 14-3-3. This interaction negates the inhibitory effect of BAD on BCL-2, thereby enhancing its anti-apoptotic properties. Additionally, AKT stabilizes protein complexes that suppress the activation of CASP-9, thereby effectively inhibiting the intrinsic apoptotic pathway [27]. IGF-1 impedes apoptosome formation by preventing the release of cytochrome c through BCL-2 stabilization and BAX inhibition. This cascade halts the activation of CASP-3, an executioner CASP responsible for the proteolysis of cellular proteins during programmed cell death [9].

### Comparative insights from other studies

The effect of IGF-1 on the apoptotic pathway is highly dose-dependent, revealing a complex balance between its protective and potentially detrimental functions. As a general mechanism, the IGF-1 signaling pathway is well-established for its protective role. A review by Hu *et al*. [35] notes that IGF-1 signaling is essential for protecting mitochondria from apoptosis, which includes stabilizing mitochondrial membrane potential and reducing ROS damage.

However, this protective mechanism is optimal only within a specific concentration range, and high concentrations can reverse this effect at the genetic level. This dose-dependency was investigated by Dai *et al*. [24], who studied the effects of varying IGF-1 concentrations on the mRNA expression of apoptosis-related genes *Bax*, *Bcl2*, and *CASP-3* in cultured mouse follicles. Their findings showed that a high concentration (50 ng/mL) significantly increased the gene expression of pro-apoptotic *Bax* and *CASP 3* while decreasing the expression of anti-apoptotic *Bcl2* [24]. This resulted in a significantly lower (i.e., worse) *Bcl2/Bax* mRNA ratio, suggesting that high IGF-1 concentrations impair pre-antral follicle function by promoting apoptosis at the transcriptional level. This aligns with other reports that follicles showed signs of degeneration at 100 ng/mL IGF-1 and that higher IGF-1 concentrations (100 ng/mL) promote apoptosis in bovine GCs.

Crucially, our findings on key downstream markers strongly suggest that the PI3K/AKT signaling pathway mediates the observed dose-dependent effects on both redox balance and apoptosis, a connection that is particularly underexplored in caprine reproductive biology. Significant upregulation of antioxidant defenses (SOD-1 and GSH) and potentiation of the anti-apoptotic protein BCL-2 are hallmark downstream effects of PI3K/AKT activation. The optimal balance of anti-apoptotic signaling (peak BCL-2) and minimal cell stress (low BAX and MDA) at 50 ng/mL suggests that this concentration achieves the most effective PI3K/AKT signaling without overstimulating detrimental metabolic processes. Although this pathway is well characterized in other species, our study provides species-specific evidence confirming its central role in regulating oocyte health during IVM in goats.

### Species-specific differences and literature comparison

A concentration of 50 ng/mL IGF-1 appeared to be more effective, owing to its synergistic action with FSH in inducing GC differentiation by modulating FSH receptor expression. The IGF system plays a crucial role in maintaining adequate FSH receptor levels, which are necessary for gonadotropin responsiveness. Low FSH receptor expression is associated with reduced follicular growth. Moreover, IGF-1 activation of the PI3K/AKT pathway and subsequent suppression of CASP activity mitigate mitochondrial stress-induced apoptosis and various intracellular signals [36]. These apoptotic pathways are integral to mitochondrial function in oocytes and CCs. Other growth factors, such as GH and its receptor, contribute to somatic growth responses, including cell proliferation, differentiation, division, and survival. They target mitochondrial function by activating the janus kinase 2/signal transducer and activator of transcription 5 pathway through endocrine and paracrine/autocrine mechanisms and can regulate glucose and lipid metabolism by stimulating multiple pathways, including PI3K/AKT, MAPK, and phospholipase C [21, 36].

The optimal IGF-1 concentration and specific effects on oocyte maturation and survival appear to be highly dependent on the species and measured endpoint. Another study in goats by Gao-Ping *et al*. [12] reported that a lower concentration of 20 ng/mL was superior for improving the final nuclear maturation rate and subsequent blastocyst development. This suggests that the ideal dose for achieving molecular stability may differ from that required for completing meiotic progression. The species-specific nature of this response is further highlighted in porcine models; Pereira *et al*. [13] found that a higher concentration of 100 ng/mL, while not improving the maturation of fresh oocytes, significantly enhanced the survival of vitrified-warmed oocytes by reducing the expression of the pro-apoptotic gene *BAX*. Collectively, these studies indicate that while moderate IGF-1 supplementation is beneficial, the precise concentration must be tailored to the specific biological goal, whether it is preserving molecular integrity.

## CONCLUSION

This study demonstrated that IGF-1 supplementation during IVM of caprine oocytes induced a concentration-dependent modulation of oxidative stress and apoptosis. Among the tested concentrations (0, 50, 100, and 150 ng/mL), the 50 ng/mL IGF-1 group (T1) exhibited the most favorable molecular profile, characterized by elevated BCL-2 and cytochrome c expression and low BAX activation, indicating optimal anti-apoptotic balance. The 100 ng/mL group (T2) achieved maximal SOD-1 and GSH levels, indicating strong antioxidant activation, whereas the 150 ng/mL group (T3) displayed increased MDA and BAX expression, suggesting excessive oxidative stress and initiation of apoptosis. These findings confirm that moderate IGF-1 concentrations enhance redox stability and mitochondrial integrity, while higher concentrations disrupt this balance and impair oocyte quality.

The observed responses can be attributed to IGF-1-mediated activation of the PI3K/AKT signaling pathway, which promotes antioxidant enzyme expression, maintains mitochondrial membrane potential, and suppresses CASP-dependent apoptosis. Moderate IGF-1 doses likely optimized PI3K/AKT phosphorylation, upregulated γ-GCS for GSH biosynthesis, and stabilized BCL-2 activity, whereas supraphysiological doses may have overstimulated cellular metabolism, leading to ROS accumulation and mitochondrial dysfunction. This biphasic behavior highlights the importance of precise dose administration for achieving the desired cytoprotective and anti-apoptotic outcomes during IVM.

The results provide a biochemical foundation for optimizing IVM media formulations in goats and potentially other small ruminants. A supplementation level of approximately 50 ng/mL IGF-1 is recommended to balance oxidative defense and cell-survival signaling without inducing stress. Applying this optimized concentration in ART programs, such as oocyte banking, *in vitro* fertilization, and embryo production, could improve developmental competence, reduce variability, and lower culture costs by avoiding unnecessary growth-factor excess. Moreover, the protocol supports genetic conservation of indigenous breeds, such as the Kacang goat, by improving oocyte utilization efficiency in tropical livestock systems.

This study is among the first to integrate biochemical (ELISA-based) and immunocytochemical assessments of both oxidative and apoptotic markers in caprine oocytes. The multi-parameter approach, linking SOD-1, GSH, and MDA with BAX, BCL-2, and cytochrome c, provides a comprehensive mechanistic map of IGF-1’s cellular effects. The experimental design, including replicate analyses and controlled dosing, adds strong internal validity and reproducibility. Furthermore, the study provides species-specific molecular evidence that distinguishes the Kacang goat response from those of bovine and porcine models.

Despite its novelty, this study has certain limitations. It primarily focused on molecular markers of oocyte quality and did not extend to functional endpoints, such as nuclear maturation rate, fertilization, or embryonic development. The activation of the PI3K/AKT pathway was inferred from downstream markers rather than confirmed by direct assays, such as Western blotting of phosphorylated AKT. As the experiment was limited to a single goat breed, inter-breed variability cannot be excluded. Broader validation across breeds and reproductive environments is warranted.

Future research should couple molecular analyses with developmental competence testing to establish a quantitative link between redox/apoptotic balance and fertilization or blastocyst yield. Investigations integrating growth-factor synergy (e.g., IGF-1 with FSH, EGF, or melatonin) may reveal additive or protective effects that enhance IVM outcomes. Omics-based studies, such as transcriptomic and proteomic profiling, could further elucidate the molecular networks modulated by IGF-1. Translational exploration of these optimized concentrations in cryopreservation, embryo transfer, and interspecies somatic cell nuclear transfer could also extend the relevance of this work to broader ART applications.

IGF-1 exhibits biphasic effects on caprine oocyte physiology, enhancing antioxidant and anti-apoptotic mechanisms at moderate concentrations, while inducing oxidative and apoptotic stress at higher concentrations. The 50 ng/mL concentration proved to be the most effective for maintaining mitochondrial stability and cellular homeostasis. These results provide a molecular blueprint for refining goat IVM protocols, improving oocyte quality, and advancing species-specific reproductive biotechnology. By establishing the optimal IGF-1 threshold, this study lays the groundwork for sustainable genetic preservation and reproductive efficiency in small-ruminant livestock systems.

## DATA AVAILABILITY

All the generated data are included in the manuscript.

## AUTHORS’ CONTRIBUTIONS

WW and EML: Conceptualization, validation, investigation, visualization, supervision, methodology, formal analysis, and drafted the manuscript. ND and VFH: Validation, investigation, and methodology. AA, WNFBWJ, and SU: Visualization, study supervision, and methodology. DYK and ZS: Visualization and drafted and revised the manuscript. All authors have read and approved the final version of the manuscript.

## References

[1] Neirijnck Y, Papaioannou M.D, Nef S (2019). The insulin/IGF system in mammalian sexual development and reproduction. Int. J. Mol. Sci.

[2] Barros V.R.P, Monte A.P.O, Santos J.M.S, Lins T.L.B.G, Cavalcante A.Y.P, Gouveia B.B, Müller M.C, Oliveira J.L, Donfack N.J, Araújo V.R, Matos M.H.T (2020). Melatonin improves development, mitochondrial function and promotes the meiotic resumption of sheep oocytes from *in vitro* grown secondary follicles. Theriogenology.

[3] Bøtkjær J.A, Pors S.E, Petersen T.S, Kristensen S.G, Jeppesen J.V, Oxvig C, Andersen C.Y (2019). Transcription profile of the insulin-like growth factor signaling pathway during human ovarian follicular development. J. Assist. Reprod. Genet.

[4] Mondal S, Mor A, Reddy I.J, Nandi S, Parameswaragupta P.S (2015). Effect of fibroblast growth factor 2 (FGF2) and insulin transferrin selenium (ITS) on *in vitro* maturation, fertilization and embryo development in sheep oocytes. Braz. Arch. Biol. Technol.

[5] Ipsa E, Cruzat V.F, Kagize J.N, Yovich J.L, Keane K.N (2019). Growth hormone and insulin-like growth factor action in reproductive tissues. Front. Endocrinol (Lausanne).

[6] Dai S, Di Z, Li N, Zeng S (2023). Optimization of recovery and maturation methods for cumulus-oocyte complexes in jennies. Reprod. Domest. Anim.

[7] Yang L, Chen Y, Liu Y, Xing Y, Miao C, Zhao Y, Chang X, Zhang Q (2021). The role of oxidative stress and natural antioxidants in ovarian aging. Front. Pharmacol.

[8] Barrera S.S, Naranjo-Gomez J.S, Rondón-Barragan I.S (2023). Thermoprotective molecules:Effect of insulin-like growth factor type I (IGF-1) in cattle oocytes exposed to high temperatures. Heliyon.

[9] Javvaji P.K, Dhali A, Francis J.R, Kolte A.P, Roy S.C, Selvaraju S, Mech A, Sejian V (2021). IGF-1 treatment during *in vitro* maturation improves developmental potential of ovine oocytes through the regulation of PI3K/Akt and apoptosis signaling. Anim. Biotechnol.

[10] Widjiati W, Darsini N, Hendrawan V.F, Taqwa S.F, Shabira Z, Kurniawati D.Y (2025). Post-warming quality of goat oocytes under heat shock stress:A study of the maturation rate, heat shock protein-70, adenosine triphosphate, and glutathione levels. Vet. World.

[11] Widjiati W, Hestianah E.P, Luqman E.M, Taqwa S.F, Caesar J, Shabira Z, Mega R.L (2024). Effect of heat shock through MDA and 8-OHdG levels of post-thawing goat oocytes. Int. J. Vet. Sci.

[12] Gao-Ping Z, Pei-Xin S, Qing C, Zi-Xin W, Yun-Xia L, Li-Xia Z, Wei S, Meng W, Si-Qin B, Gui-Fang C, Xi-He L (2023). Effects of insulin-like growth factor 1, leukemia inhibitory factor, and basic fibroblast growth factor on goat oocyte maturation and early embryonic development *in vitro*. Small Rumin. Res.

[13] Pereira B.A, Zangeronimo M.G, Castillo-Martín M, Gadani B, Chaves B.R, Rodríguez-Gil J.E, Bonet S, Yeste M (2019). Supplementing maturation medium with insulin growth factor i and vitrification-warming solutions with reduced glutathione enhances survival rates and development ability of *in vitro* mature vitrified-warmed pig oocytes. Front. Physiol.

[14] Kasman A.A.M.N, Santoso B, Widjiati W (2020). The effect of vitrification after warming on the expressions of p38, CDK1, and cyclin B in immature goat oocytes followed by *in vitro* maturation. Vet. World.

[15] De Figueiredo C.S, Raony I, Medina S.V, De Mello Silva E, Dos Santos A.A, Giestal-De-Araujo E (2022). Insulin-like growth factor-1 stimulates retinal cell proliferation via activation of multiple signaling pathways. Curr. Res. Neurobiol.

[16] Ianza A, Sirico M, Bernocchi O, Generali D (2021). Role of the IGF-1 axis in overcoming resistance in breast cancer. Front. Cell Dev. Biol.

[17] Yang S, Yang Y, Hao H, Du W, Pang Y, Zhao S, Zou H, Zhu H, Zhang P, Zhao X (2022). Supplementation of EGF, IGF-1, and connexin 37 in IVM medium significantly improved the maturation of bovine oocytes and vitrification of their IVF blastocysts. Genes (Basel).

[18] Jiang Q, Lou K, Hou L, Lu Y, Sun L, Tan S.C, Low T.Y, Kord-Varkaneh H, Pang S (2020). The effect of resistance training on serum insulin-like growth factor 1 (IGF-1):A systematic review and meta-analysis. Complement. Ther. Med.

[19] Gualtieri R, Kalthur G, Barbato V, Di Nardo M, Adiga S. K, Talevi R (2021). Mitochondrial dysfunction and oxidative stress caused by cryopreservation in reproductive cells. Antioxidants (Basel).

[20] Poudel S.B, Dixit M, Neginskaya M, Nagaraj K, Pavlov E, Werner H, Yakar S (2020). Effects of GH/IGF on the aging mitochondria. Cells.

[21] Lyons M, Coleman S, Riis S, Favre C, O'Flanagan C.H, Zhdanov A.V, Papkovsky D.B, Hursting S.D, O'Connor R (2017). Insulin-like growth factor 1 signaling is essential for mitochondrial biogenesis and mitophagy in cancer cells. J. Biol. Chem.

[22] Arjunan A, Sah D.K, Woo M, Song J (2023). Identification of the molecular mechanism of insulin-like growth factor-1 (IGF-1):A promising therapeutic target for neurodegenerative diseases associated with metabolic syndrome. Cell Biosci.

[23] Martins R, Lithgow G.J, Link W (2016). Long live FOXO:Unraveling the role of FOXO proteins in aging and longevity. Aging Cell.

[24] Dai S, Zhang H, Yang F, Shang W, Zeng S (2022). Effects of IGF-1 on the three-dimensional culture of ovarian preantral follicles and superovulation rates in mice. Biology (Basel).

[25] Soto-Heras S, Paramio M.T (2020). Impact of oxidative stress on oocyte competence for *in vitro* embryo production programs. Res. Vet. Sci.

[26] Ge L, Liu S, Rubin L, Lazarovici P, Zheng W (2022). Research progress on neuroprotection of insulin-like growth factor-1 towards glutamate-induced neurotoxicity. Cells.

[27] Jung N.H, Kim S.H, Kim D.S, Yoon J.T (2020). Study on the *in-vitro* culture method for normal embryonic cell development of porcine parthenogenetic embryos. J. Anim. Reprod. Biotechnol.

[28] Choi J.Y, Jo M.W, Lee E.Y, Yoon B.K, Choi D.S (2010). The role of autophagy in follicular development and atresia in rat granulosa cells. Fertil. Steril.

[29] Morohaku K, Tanimoto R, Sasaki K, Kawahara-Miki R, Kono T, Hayashi K, Hirao Y, Obata Y (2016). Complete *in vitro* generation of fertile oocytes from mouse primordial germ cells. Proc. Natl. Acad. Sci. U. S. A.

[30] Llavanera M, Mateo-Otero Y, Bonet S, Barranco I, Fernández-Fuertes B, Yeste M (2020). The triple role of glutathione S-transferases in mammalian male fertility. Cell. Mol. Life Sci.

[31] García-Mato Á, Cervantes B, Rodríguez-De La Rosa L, Varela-Nieto I (2023). IGF-1 controls metabolic homeostasis and survival in HEI-OC1 auditory cells through AKT and mTOR signaling. Antioxidants (Basel).

[32] Hakuno F, Takahashi S.I (2018). IGF1 receptor signaling pathways. J. Mol. Endocrinol.

[33] Riis S, Murray J.B, O'Connor R (2020). IGF-1 signalling regulates mitochondria dynamics and turnover through a conserved GSK-3b-Nrf2-BNIP3 pathway. Cells.

[34] Jung H.J, Suh Y (2015). Regulation of IGF-1 signaling by microRNAs. Front. Genet.

[35] Hu B, Li H, Zhang X (2021). A balanced act:The effects of GH-GHR-IGF1 axis on mitochondrial function. Front. Cell Dev. Biol.

[36] Aguirre G.A, De Ita J.R, De La Garza R.G, Castilla-Cortazar I (2016). Insulin-like growth factor-1 deficiency and metabolic syndrome. J. Transl. Med.

